# Novel Lipophilic Lanthanide Bis-Phthalocyanines Functionalized by Pentadecylphenoxy Groups: Synthesis, Characterization and UV-Photostability

**DOI:** 10.3390/molecules170910738

**Published:** 2012-09-07

**Authors:** Rudolf Słota, Gabriela Dyrda, Maria Hofer, Giuseppe Mele, Ermelinda Bloise, Roberta del Sole

**Affiliations:** 1 Faculty of Chemistry, Opole University, ul. Oleska 48, 45-052 Opole, Poland; 2 Department of Engineering for Innovation, University of Salento, Via Arnesano, 73100 Lecce, Italy

**Keywords:** lanthanide bis-phthalocyanines, cardanol, UV-Vis spectroscopy, photostability

## Abstract

Novel sandwich-type phthalocyanines containing a rare earth metal core (Pr, Nd, Eu–Lu) and macrocycles peripherally substituted by pentadecylphenoxy groups were synthesized using a cardanol-based phthalonitrile precursor and the respective lanthanide acetate. Additionally, the metal free-base analog compound was studied for comparison. The purified reaction products were all found to be thick and viscous substances at room temperature, showing liquid crystalline behavior with a distinct increase in fluidity at ca. 40 °C. The complexes are readily soluble in chloroalkyl solvents and dissolve fairly well in DMF with some tendency to form aggregates. Besides they are strongly hydrophobic and reveal a peculiar affinity for lipophilic media. The compounds have been characterized by UV-Vis (absorption and emission), FTIR, MS and DSC methods. Photochemical activity in the liquid phase (dimethylformamide, dichloromethane, mineral oil) and the degree of photodegradation demonstrated under constant UV-irradiation (λ = 352 nm) have been analyzed and discussed in terms of photostability.

## 1. Introduction

The first successful synthesis of lanthanide phthalocyanine double-deckers reasonably documented by spectral data was simultaneously reported by the two independent Russian research groups of Plyushev and Kirin in the 1960s [1,2]. Soon after, it was found these peculiar complexes possessed very interesting electronic and optoelectronic properties, unique among the phthalocyanine family. Besides, they manifested a specific chemistry toward diverse electron acceptor and/or donor species and displayed unusual photochemical activity. Therefore, the rare earth phthalocyanine derivatives have been placed among the highest ranked molecular materials considered important for hi-tech applications [3]. A comprehensive overview related to these issues has been presented in some compilations, e.g., [4,5] whereas diverse synthesis methods have also been summarized and discussed in a dedicated review [6].

**Figure 1:**
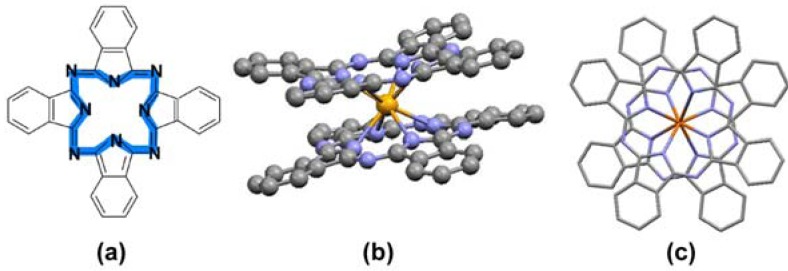
(**a**) Phthalocyanine macrocycle with the marked π-electronic core; (**b**) Sandwich structure of LnPc_2_; (**c**) Top view of the sandwich molecular system.

**Figure 2:**
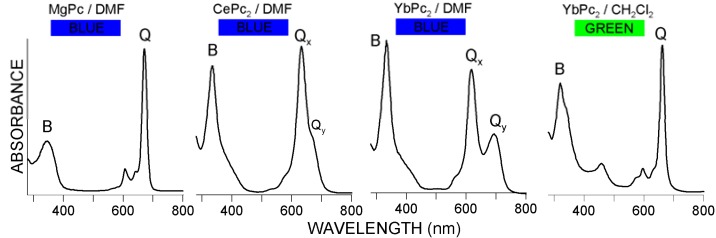
Electronic absorption spectra of diverse metallophthalocyanines in DMF and dichloromethane; note the differences in position and shape of the B and Q bands, as well as the solution color.

The molecular setup in bis-phthalocyanines (LnPc_2_) offers additional possibilities relative to the typical ′mono′ compounds (MPc). Two macrocycles coordinated by the rare earth metal template (Ln) create a very stable π-electronic bonding system (Figure 1) which is considered a neutral sandwich radical having the electronic charge delocalized over both the Pc units [5]. Such an arrangement allows for changing the distribution of electronic density within the cores of the particular phthalocyanine moieties whereas the molecular structure of the sandwich complex remains intact. Hence, the spectral diversity displayed in Figure 2 is characteristic exclusively for the LnPc_2_ systems and not for the MPc ones. This fact is believed to reflect some kind of specific flexibility of the electronic clouds of the Pc rings in LnPc_2_, manifested as a result of internal and/or external polarization effects [5,7,8].

Peripherally functionalized LnPc_2_’s represent an interesting variety of phthalocyanines because of additional unique features they may demonstrate depending on the substituent(s) attached to the benzene ring(s) of the macrocycle, e.g., water solubility, hydrophobicity, enhanced activity, *etc*. [5]. Compounds heavily substituted by long-chain aliphatic groups reveal liquid crystalline behavior which has been well documented in the literature reported so far [9,10,11,12,13,14,15]. Such phthalocyanines, due to molecular self-assembly, produce a kind of discotic mesophases displaying a range of interesting optoelectronic properties including electrochromism [12]. The bulky sterically hindering groups linked to the macrocycles may contain sulfur atoms (e.g., hexylthio- [14]) as well as both aromatic (e.g., phenoxy- [13]) and non-aromatic (e.g., cyclohexyl- [16]) rings.

**Figure 3:**
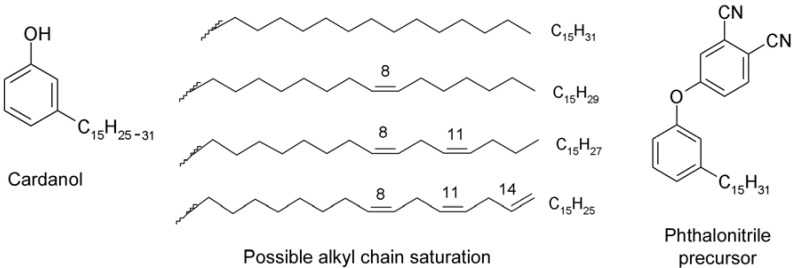
Cardanol and the phthalonitrile precursor.

Cardanol is an important natural and renewable organic raw material obtained as a by-product from the cashew nut agroindustry [17]. The chemistry of cardanol is becoming a stimulating area in academic and industrial research particularly with a view to preparation of new eco-friendly fine chemicals and functional organic materials [18,19]. The peculiar properties of cardanol itself and its derivatives, such as relatively high solubility in non-polar media and good processability, follow from the presence of the C_15_ alkyl chain at the *meta* position of the phenolic ring (Figure 3).

The first phthalocyanines containing cardanol-derived functional groups including a C_15_ alkyl chain were synthesized in the early 2000s [20]. These were the metal-free base (H_2_Pc) and complexes of the MPc (mono) type, where M was Ni, Cu, Zn and Pd. The synthesis was performed in a liquid medium using a pentadecylphenoxy-phthalonitrile precursor shown in Figure 3 (for more details see Ref. [20]). Surprisingly, the reaction products were all found to melt already at about 40 °C, which was unusual when compared with typical solid crystalline phthalocyanine materials. This particular feature, similar to that demonstrated by discotic liquid crystalline phases, along with a high solubility in organic solvents may be important for diverse application purposes. 

In the search of novel molecular modifications of the double-decker lanthanide phthalocyanines the pentadecylphenoxy-functionalized derivatives have seemed particularly interesting. Generally, because they were expected to display similar chemical and photochemical properties as their non-substituted counterparts and additionally some features normally not revealed by the typical LnPc_2_ compounds, such as extremely low melting and/or softening point and enhanced affinity to fatty media (lipophilicity). Besides, the effect of the pentadecylphenoxy- groups upon the activity and stability of the coupled macrocycle system was considered important and crucial to phthalocyanine materials science. 

## 2. Results and Discussion

### 2.1. Synthesis and General Properties

**Figure 4:**
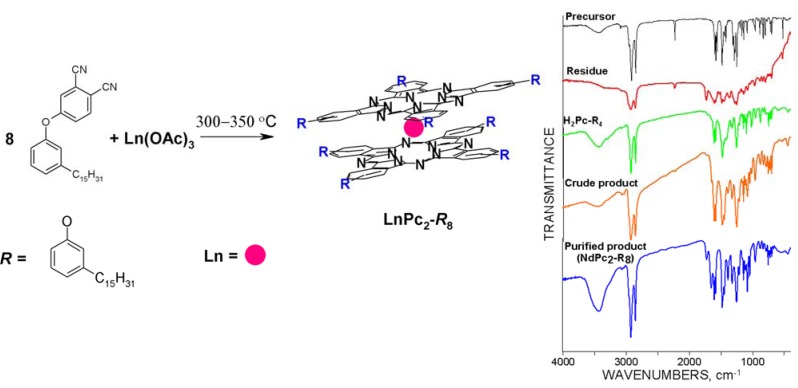
Synthesis of lanthanide bis-phthalocyanines functionalized by pentadecyl-phenoxy-groups, featuring the hypothetical molecular structure of the target complex. On the right, FTIR spectra illustrating the composition of the post-reaction system.

All syntheses were performed based on Kirin’s method [2], with some procedural modifications according to Słota and co-workers [21] (see Experimental Section), using the same phthalonitrile precursor as reported above (Figure 3, [20]). The reaction scheme is shown in Figure 4, supplemented by FTIR spectra reflecting the effectiveness of the applied method.

**Figure 5:**
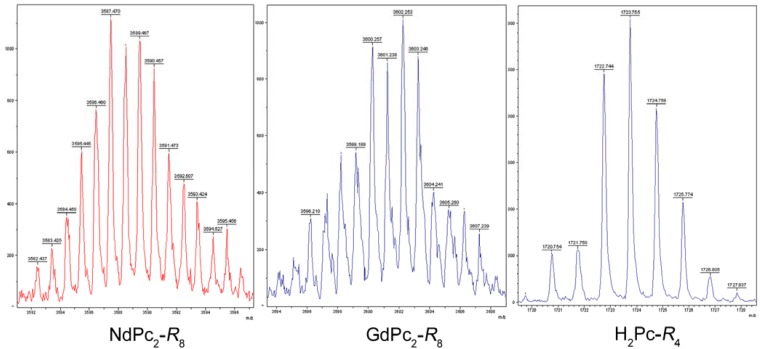
Mass spectra (MALDI-ToF) of the novel bis-phthalocyanines and the metal-free base phthalocyanine; spectra patterns of NdPc_2_-*R*_8_, GdPc_2_-*R*_8_ and H_2_Pc-*R*_4_ have been shown to confirm the molecular structure of the synthesized complexes (see Table 1).

**Figure 6:**
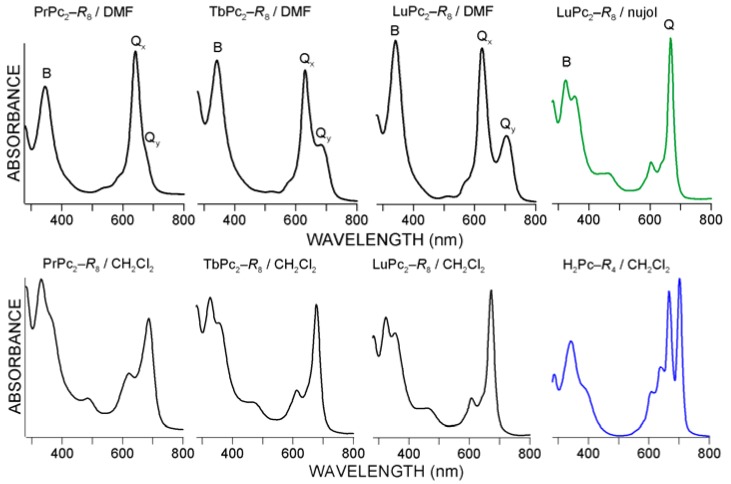
UV-Vis electronic absorption spectra-types registered for LnPc_2_ in DMF and nujol (up) and in dichloromethane (down) including H_2_Pc-*R*_4_; for λ_max_ details see Table 2.

**Figure 7:**
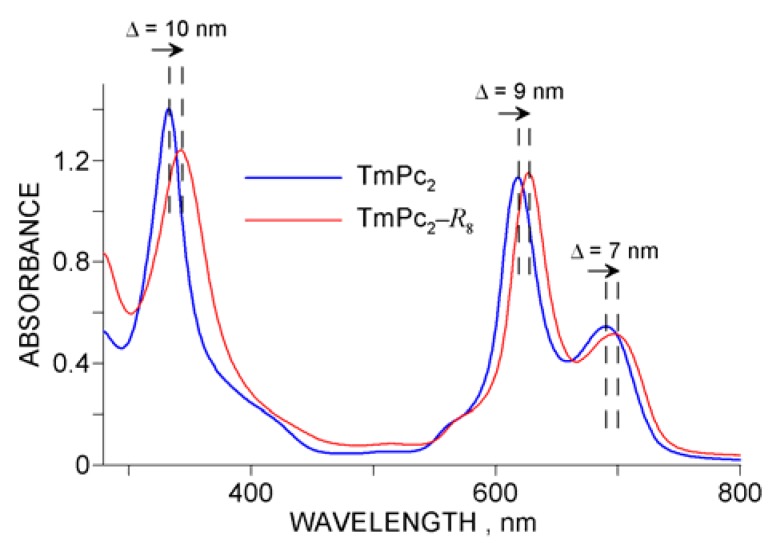
Comparison of UV-Vis spectra of TmPc_2_ and TmPc_2_-*R*_8_ in DMF; ∆ values reflect the effect of pentadecylphenoxy- peripheral substitution of the phthalocyanine macrocycles.

**Figure 8:**
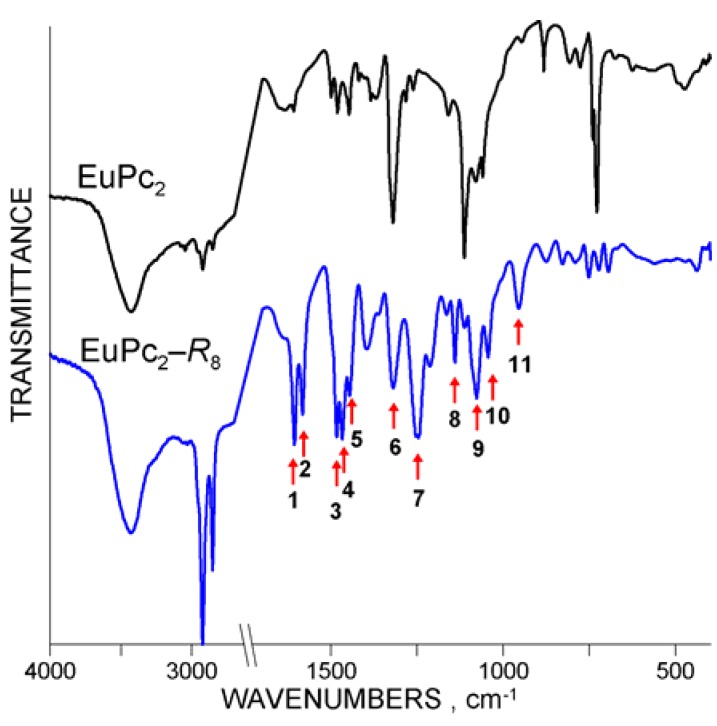
Effect of pentadecylphenoxy-substitution in EuPc_2_ displayed in the FTIR spectra (note the X-axis has been broken between 1700 and 2700 cm^−1^ for brevity).

The molecular structure of the novel complexes was generally confirmed by mass spectroscopy results obtained using the MALDI-ToF method (Figure 5, Table 1), and supported both by the UV-Vis (Figure 6 and Figure 7, Table 2) and FTIR (Figure 8, Table 3) spectra. As expected, the reaction yielded sandwich compounds with the macrocycles peripherally functionalized by eight pentadecylphenoxy- groups (or four such groups in the case of H_2_Pc). The *m/z* data reported in Table 1 fit very well the calculated molecular mass and hence the general formula of these novel phthalocyanine analogues could be assumed as LnPc_2_-*R*_8_ with a hypothetical molecular setup like that suggested in Figure 4. Obviously, there are still many questions concerning the structural isomers possible, as well as the spatial orientation of the alkyl chains and so on, however these issues did not fit the scope of this study and hence will be addressed in a forthcoming project.

**Table 1:** Mass spectra *m/z* results (MALDI-ToF) acquired for the novel bis-phthalocyanines of Nd and Gd, and the metal-free base phthalocyanine and the calculated molecular mass (M). (For more data see Supplementary Information appendix).

Compound	M (calc.)	*m/z* (exp.)
H_2_Pc-*R*_4_	1726	1722–1726
NdPc_2_-*R*_8_	3593	3585–3593
GdPc_2_-*R*_8_	3605	3599–3604

**Table 2:** Peak position of the B (Soret) and Q bands (λ_max_, nm) in the UV-Vis spectra of LnPc_2_-*R*_8_. Values of Q_y_ for Pr–Tb were determined using the Spectra Manager V.2 analytical software.

	Pr	Nd	Eu	Gd	Tb	Dy	Ho	Er	Tm	Yb	Lu	H_2_Pc
						DMF						
λ (B)	346	343	344	343	342	344	342	342	343	342	341	-
λ (Q_x_)	641	640	633	632	631	629	629	628	627	626	624	-
λ (Q_y_)	675	676	681	682	683	687	694	695	697	702	704	-
**Dichloromethane**												
λ (B)	330	328	326	327	326	327	325	325	325	325	324	342
λ (Q)	686	687	681	678	677	675	675	678	673	672	672	666/701
**Nujol**												
λ (B)	333	328	327	327	327	327	326	327	326	325	324	342
λ (Q)	681	682	677	674	673	671	671	678	669	668	667	664/703

**Table 3:** Vibration frequencies (cm^−1^) registered in the FTIR spectra of EuPc_2_-*R*_8_ corresponding to the numbers assigned to the chosen bands in Figure 8.

Wavenumbers, cm^−1^											
	**1**	**2**	**3**	**4**	**5**	**6**	**7**	**8**	**9**	**10**	**11**
Eu	1606	1582	1483	1467	1446	1320	1247	1140	1077	1044	955

The crude reaction product always contained some quantity of the metal-free phthalocyanine (H_2_Pc-*R*_4_), which had been separated during the extraction process performed in dimethylformamide (DMF) since it was insoluble in this solvent. Portions of this compound were collected from different syntheses and purified by column chromatography (Al_2_O_3_, eluent CH_2_Cl_2_), and further used as a comparative material relative to the target LnPc_2_-*R*_8_ complexes. 

At room temperature, the purified complexes (and also the metal-free base) are dark navy-blue non-crystalline materials characterized by a semi-liquid phase structure of rather high viscosity showing a rapid increase in fluidity at about 40 °C, as observed elsewhere for diverse phthalocyanine discogens [11]. Undoubtedly, such behavior must be induced by the presence of the C_15_-alkylo-aromatic substituents at the macrocycle's periphery and hence the novel LnPc_2_-*R*_8_ compounds may be added to the expanding list of discotic liquid crystalline phthalocyanine systems.

The novel LnPc_2_-*R*_8_ compounds dissolve fairly well in DMF, with some susceptibility to form aggregates; however they are particularly readily soluble in chloroalkyl solvents (e.g., CH_2_Cl_2_). After drying out, the thin film left on a glass substrate is uniform and clearly transparent, ranging in color from practically invisible through greenish-blue to marine-blue, unlike the case of typical LnPc_2_’s. In the liquid phase the studied phthalocyanines proved chemically stable, at least as long as stored in dark. Moreover, solutions of these complexes are also possible in nujol (mineral oil). This latter finding has revealed another very interesting feature manifested by the pentadecylphenoxy phthalocyanine derivatives, namely their affinity for fatty media. Besides, they were found to be highly hydrophobic and in an independent preliminary study we have confirmed their solubility also in a phospholipid environment, successfully using the same electron spin resonance technique (ESR) as applied in our recent study dedicated to lipophilic metalloporphyrins [22]. 

### 2.2. UV-Vis Spectra

Since the re-discovery of phthalocyanines for practical engineering and technology applications in the early 1930s, UV-Vis spectroscopy has been considered the principal analytical method crucial to explore the physicochemical nature of these materials. Like the typical rare earth metal bis-phthalocyanines [5,21], a very strong absorption of photons from the UV range as well as from the red end of the Vis spectrum with a molar absorptivity of about 10^5^ M^−1^ cm^−1^ characterize the optoelectronic properties revealed by the novel LnPc_2_ derivatives. The spectra reported in Figure 6 have been chosen especially to represent the peculiar features displayed by the LnPc_2_-*R*_8_ molecular systems, showing diverse possibilities relative to the incorporated Ln atom and the liquid medium used. Complete spectral data have been collected in Table 2.

As follows from Figure 2 and Figure 6, the character of the LnPc_2_ UV-Vis spectra (and hence the solution color) apparently depends on the solvent polarity, therefore the LnPc_2_ solutions are blue in the polar DMF and green both in low-polar dichloromethane and non-polar nujol [4,5].

Basically, the spectra measured for the novel compounds display a similar layout of absorption bands as in the case of typical lanthanide bis-phthalocyanines. Obviously the peripheral substitution of the macrocycles should have led to certain structural perturbations which in consequence might have affected the UV-Vis spectra, as shown in 7. In comparison with the non-functionalized LnPc_2_’s, distinct red-shifts of the relevant absorption bands can be noted, which generally correspond to the values shown for the Tm-complexes, independently of the Ln metal. Similar observations have been reported elsewhere for octadecyl-substituted LuPc_2_ [12].

The spectra shown in Figure 6 demonstrate the well-known very subtle relationship between the individual phthalocyanine macrocycles and the lanthanide metal hosted by the sandwich system [5]. In particular in DMF a distinct splitting of the Q band into two components, Q_x_ and Q_y_, can be noted. This splitting widens gradually with the increasing Ln atomic number (from 34 nm for Pr up to 80 nm for Lu, Table 2). Simultaneously, the λ_max_ peak value of the Q band decreases from Pr to Lu and the same trend is shown by the B (Soret) band. In dichloromethane and nujol a similar shift in the Q and B band position may be observed, however the Q-bands do not split. Such changes reflect the growing effect of polarizing forces inside the sandwich molecular system resulting from the contracting ionic radius of the complexed Ln^3+^ species, from Pr^3+^ (99 pm) to Lu^3+^ (86 pm). Undoubtedly, this fact could explain the differences in chemical reactivity and photoactivity of the diverse LnPc_2_ compounds. 

### 2.3. Infrared Spectra

Vibrational spectra of all studied LnPc_2_-*R*_8_ compounds, measured in the infrared range 400–4000 cm^−1^, generally display a similar pattern as that of the europium complex shown in Figure 8. Frequencies corresponding to the numbered bands have been reported in Table 3. Since the IR spectra represent a very complex layout of absorption bands due to diverse coupling effects produced by the oscillating bonding system of the both phthalocyanine macrocycles and by the pentadecylphenoxy groups, their discussion was not deemed relevant at this point and the full FTIR data with some comments have been reported in the Supplementary Information electronic file. Note however the characteristic bands in the following ranges: 2850–3000, 1582 (peak No. 2), 1445–1483 (No. 3–5) and 1247–1324 cm^−1^ (No. 6–7), which directly confirm the presence of pentadecylphenoxy-groups attached to the phthalocyanine moiety.

### 2.4. Thermal Stability

**Figure 9:**
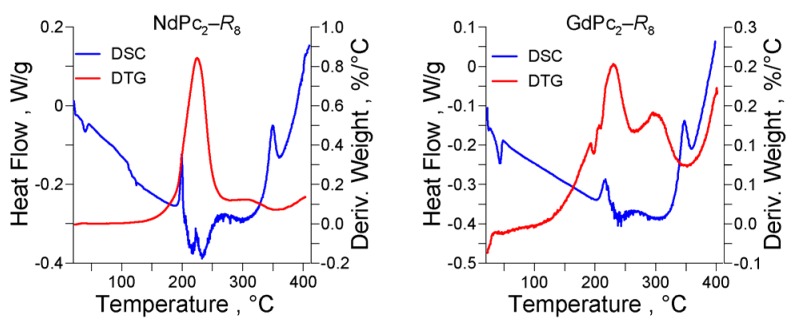
DSC and DTG curves measured for NdPc_2_-*R*_8_ and GdPc_2_-*R*_8_.

Thermal analysis revealed similar behavior on heating for all synthesized LnPc_2_-*R*_8_ complexes as demonstrated by the DSC and DTG curves presented in Figure 9. The non-crystalline quasi-liquid substances become distinctly more fluid at about 40 °C as indicated by the small endothermic peak. For the studied materials this heat-effect was detected in the range of 39 °C (Nd) and 43 °C (Gd); for the H_2_Pc it was 36 °C. Two other effects accompanied by a significant loss in mass (30–45%) appear generally between 200 °C and 240 °C which may account for the first stage of the compound’s degradation. The exothermic effect at ca. 350 °C may probably be related to the macrocycle cleavage. These observations reveal the discotic liquid crystalline nature of the novel LnPc_2_-*R*_8_ compounds and are consistent with the results reported for LuPc_2_ with n-alkyl-octasubstituted macrocycles [12,13].

The total loss in mass on heating up to 400 °C was about 60%. No distinct correlation to the Ln metal was revealed, however the compounds including the heavier lanthanides (Gd–Lu) seem somewhat more heat-resistant than those of Pr, Nd and Eu. Nevertheless, a plausible explanation of the thermal properties of LnPc_2_-*R*_8_ would have required a more profound and detailed DSC/TGA study. At this point, however, the results obtained thus far clearly indicate that the novel LnPc_2_-*R*_8_ complexes (as well as the metal-free compound) are definitely less thermally stable than their non-substituted counterparts.

### 2.5. Fluorescence Emission Spectra

The novel LnPc_2_-*R*_8_ complexes and H_2_Pc-*R*_4_ when excited by UV radiation displayed emission patterns of a complicated nature showing bands both in the blue and red range of the visible spectrum. In most cases the Q-band emission was found to be very weak due to the heavy atom effect. Since the fluorescence results seemed to be too problematic to interpret at this stage of investigation, the measured spectra have been reported and some comments given in the Supplementary Information appendix. However, the emission nature in LnPc_2_-*R*_8_ will be reconsidered in a forthcoming dedicated project.

### 2.6. Photostability in Liquid Media (DMF, Dichloromethane, Mineral Oil)

Generally, the studied LnPc_2_-*R*_8_ phthalocyanines undergo photodegradation due to absorption of photonic energy from the UV range; however, the cleavage mechanism of the macrocyclic systems clearly depends both on the Ln metal and the solvent used. It must be emphasized, however, the extremely high phototostability manifested by each of the tested compounds in mineral oil (nujol). In fact, only in the case of H_2_Pc-*R*_4_ some apparent but very slow decay was observed, whereas for LnPc_2_-*R*_8_the changes registered during the 6 h of exposure to UV radiation (λ = 352 nm, 300 μW∙cm^−2^) were found negligible. 

**Figure 10:**
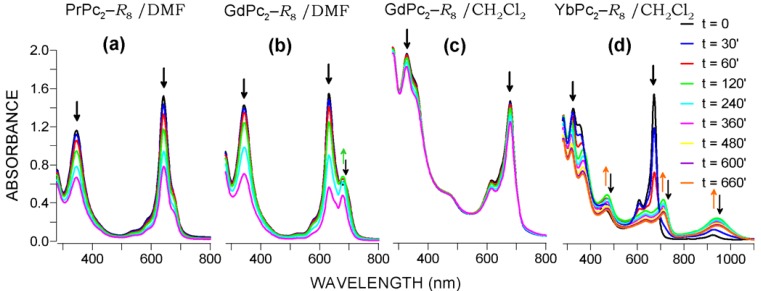
Photolysis of LnPc_2_-*R*_8_ in DMF and CH_2_Cl_2_ under constant UV irradiation (λ = 352 nm, 300 μW∙cm^−2^) featuring diverse types of reaction kinetic; total irradiation time, t = 360 min (660 min for Yb). Arrows indicate the trend of changes in the solution composition. Development of the green and orange forms is highlighted by the respective colors while their extinction is indicated by the black arrows.

In a given medium, the photolysis process basically follows a similar scheme. The apparent differences observed in the spectra of diverse compounds during irradiation, as presented in Figure 10, result only from the susceptibility of the individual molecules to modifications of electronic density distribution within the π-electronic cores which is definitely controlled by the incorporated metal (polarization effect). Redistribution of these electrons may be considered as a response of the molecular setup to some external factors (e.g., radiation, heat, reactive species) in order to preserve the sandwich system intact. Hence, we may observe the characteristic spectral changes marked in Figure 10b,d by the appropriate colored arrows, indicating the raising of the green and orange forms, respectively. Certainly, development of the highlighted absorption bands should be related to pronounced changes in distribution of electronic density within the coordination sphere. The somewhat controversial nature of the colored forms of LnPc_2_’s was commented elsewhere [5]; it was also discussed in our previous publications in terms of “molecular flexibility” revealed by the bonding system in rare earth metal bis-phthalocyanines [8,23].

The quoted green and orange forms have been detected essentially in solutions of the heavier Ln complexes. However, a thorough analysis of the relevant spectra also indicated the development of the green form in DMF solutions of the Pr and Nd compounds, but in too low concentration to be recognized in Figure 10a. This fact may have confirmed generally the same photolysis mechanism in DMF for all LnPc_2_-*R*_8_ systems studied. Nevertheless, in dichloromethane (Figure 10d) the orange form emerged exclusively for the complexes of the Tb–Lu series. Thus, the photodegradation for the lighter Ln elements proceeds according to a typical one-step mechanism, represented by the changes seen in Figure 10c.

**Figure 11:**
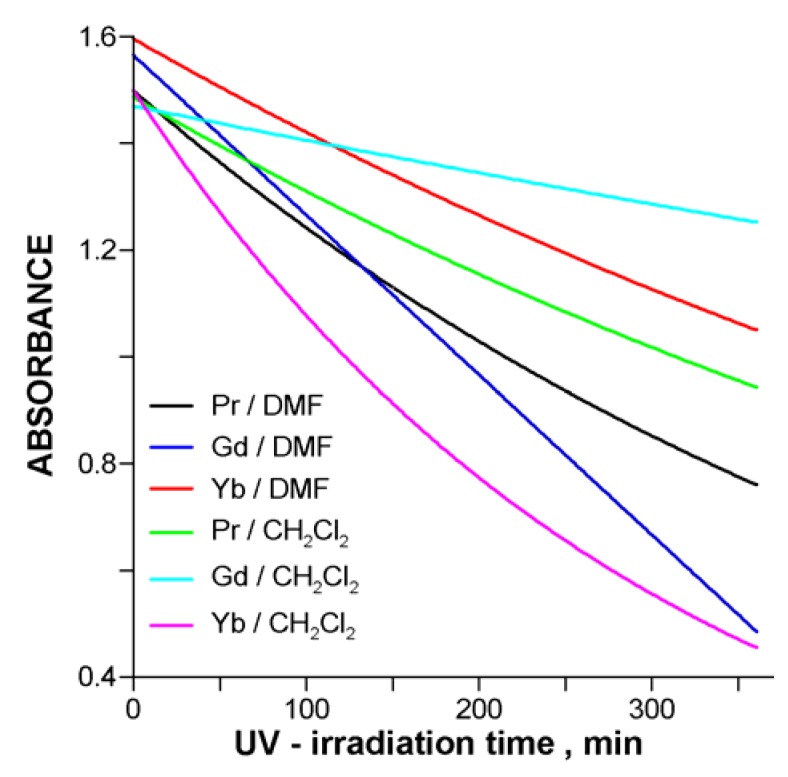
Representative kinetic curves illustrating the UV-photolysis of LnPc_2_-*R*_8_ in DMF and CH_2_Cl_2_.

The character of the Q-band extinction revealed in the spectra of the heavier Ln compounds indicates for a set of two successive reactions (A→B→C), *i.e.*, phototransformation (A→B) followed by degradation of the molecular system (B→C). Both the initial form (A) and the consecutive one (B) decompose gradually, as resulted from the analysis of kinetic data, and which may also be seen in Figure 10b,d. A closer examination of the Soret range showed that the continuous decrease in absorbance of the B-band fitted well the first-order reaction law. Accordingly, we have assumed it should be correlated immediately with the decay of the phthalocyanine system, no matter which of the both forms (A or B) has momentary dominated the Q-band. Since these conclusions basically appeared reasonable and consistent with the photolysis data of the all systems studied a simple procedure based on the B-band extinction rate was employed to compare the photostability of the explored phthalocyanines. Kinetic curves representing the time-related changes in B-band absorbance of the UV-irradiated solutions, A = *f* (*t*), revealed a typical exponential character, as shown in Figure 11.

The effective rate constant, *k_e_* (min^−1^), of the photodegradation process was computed from the exponential fitting expressed by equation (1), applied to the individual kinetic data. In all cases excellent correlation coefficients showing R^2^ >0.99 were achieved: 


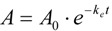
(1)


**Figure 12:**
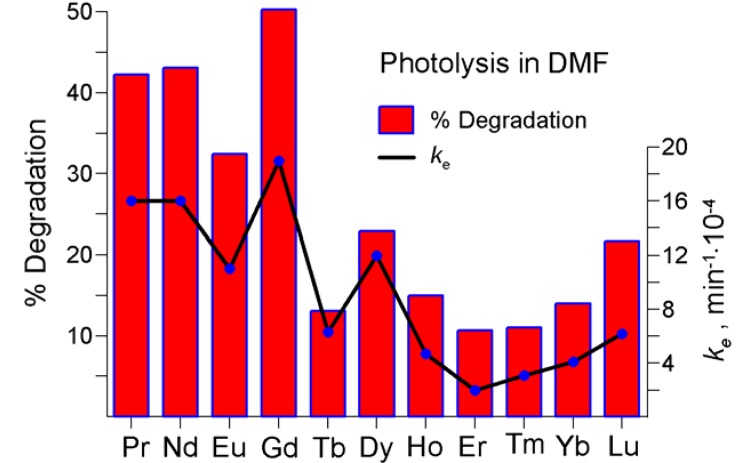
Degradation degree (%) and effective reaction rate constants, *k*_e_ (min^−1^) during the photolysis carried out in DMF (for details see Table 4).

**Figure 13:**
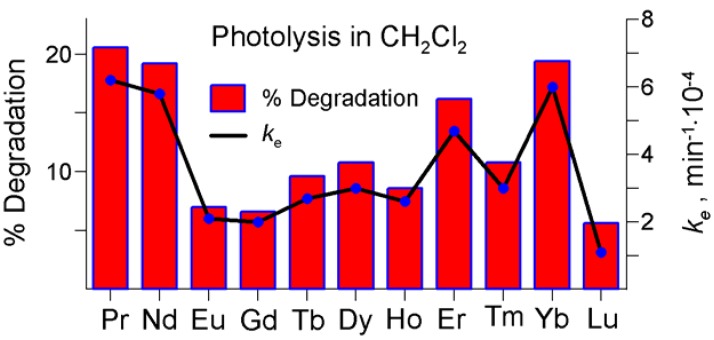
Degradation degree (%) and effective reaction rate constants, *k*_e_ (min^−1^) during the photolysis carried out in dichloromethane (for details see Table 4).

The degree of the compounds degradation due to photolysis, δ (%), was estimated from the initial and final absorbance measured at λ_max_ of the B-band, for the established irradiation time of 360 min. Values of the both parameters have been reported in Table 4 and visualized in Figure 12 and Figure 13.

The compounds explored in this study revealed diverse resistance to the UV radiation, however the metal free base phthalocyanine proved definitely unstable in dichloromethane solution (H_2_Pc-*R*_4_ is insoluble in DMF). Indeed, this fact accounts for the macrocycle-stabilizing role of the hosted metal ion.

**Table 4:** Photolysis effective rate constant, *k_e_* (min^−1^∙10^−4^) and the degree of photodegradation, δ (%) determined from kinetic curves A = *f(t)* for the B-band in DMF and dichloromethane (DCM) after 360 min of UV-irradiation. *^)^ For H_2_Pc after 135 min of UV-irradiation, *k_e_* and δ estimated for the Q-band.

	Pr	Nd	Eu	Gd	Tb	Dy	Ho	Er	Tm	Yb	Lu	H_2_Pc *^)^
*k_e_* /DMF	16	16	11	19	6.3	12	4.7	2.0	3.1	4.1	6.2	-
δ, %	42.2	43.1	32.4	50.3	13.1	22.9	15.0	10.7	11.0	14.0	21.7	-
*k_e_* /DCM	6.2	5.8	2.1	2.0	2.7	3.0	2.6	4.7	3.0	6.0	1.1	260
δ, %	20.6	19.2	7.0	6.6	9.6	10.8	8.6	16.2	10.8	19.4	5.6	91.2

Generally, photostability of the novel LnPc_2_-*R*_8_ complexes does not show a distinct correlation with the coordinated Ln atom. Interestingly, the photolysis progress demonstrated in dichloromethane was somewhat surprising, particularly when considering the relatively low reaction rates (Table 4). For the non-substituted LnPc_2_’s investigated so far, the photodegradation was always faster in dichloromethane (as well as in other alkyl-halogen solvents) than in DMF. Incidentally, the *k_e_* values displayed in dichloromethane by the pentadecylphenoxy-substituted derivatives feature a comparable order of magnitude as in the case of the non-substituted counterparts, unlike in DMF where the *k_e_* results proved considerably lower for LnPc_2_-*R*_8_ (even 100-times for Eu). Obviously, the compounds of the Ho–Lu series show reasonable photostability in DMF and this fact does comply with the arguments raised in Subsection 2.2. 

## 3. Experimental

### 3.1. General

All phthalocyanines investigated and described in this work as well as the lanthanide acetates were synthesized in the laboratory of the Faculty of Chemistry (Opole University, Opole, Poland) by R. Słota and the phthalonitrile precursor was synthesized by G. Mele (University of Salento, Lecce, Italy). Pure grade solvents (DMF, dichloromethane and nujol) were purchased from Sigma-Aldrich Poland and used as supplied. 

*Elemental analysis:* EA 1108 CHNS-O analyzer (Fisons Instruments, Ipswich, UK); results of %C, %H and %N representative for all LnPc_2_-*R*_8_ compounds have been reported using the neodymium complex (anal/calcd; C: 75.0/77.5, H: 8.2/8.7, N: 5.5/6.2) as an example and additional comments provided in the Supplementary Information appendix. 

*Mass spectroscopy:* Reflex IV spectrometer (Bruker Daltonik, Bremen, Germany), Flex Control 2.4 software; MALDI-ToF method, α-cyano-4-hydroxycinnamic acid (saturated solution in water/acetonitrile/TFA 66.9/33/0.1) as ionization adjuvant.

*UV-Vis measurements:* JASCO V-670 Spectrophotometer (UV-Vis-NIR, Jasco), 1 cm quartz cuvettes, Jasco Spectra Manager V.2 software.

*FTIR measurements:* Spectrometers PV 9800 (Philips); Nexus (Thermo Nicolet); KBr pellets.

*Thermal analysis:* TA-1 (Mettler); DSC/TGA curves measured under constant nitrogen flow.

*Fluorescence measurement:* Spectrofluorimeter Hitachi F-7000 (Hitachi Instrument), 1 cm quartz cuvettes.

*UV irradiation:* UV lamp NU-8 (Herolab, Germany), 8 W Hg-tube; λ_max_ at 352 nm. 

### 3.2. Synthetic Procedure

The phthalonitrile precursor (Figure 4) is mixed with the desired lanthanide acetate [Ln(ac)_3_∙1.5 H_2_O] in a stoichiometric molar ratio (Figure 4) and heated for 1 h in a sealed Pyrex-glass ampoule at a temperature predetermined for the individual sets of substrates; the adequate temperature range was found to be between 320 °C and 350 °C. For this reason we use a transparent glass reactor (electrically heated) which allows immediate visual monitoring of the reaction progress. The formation of the phthalocyanine complex essentially proceeds in the liquid phase, right after melting of the substrates (approx. 260 °C) which is evidenced by distinct changes in the color of the liquid. When the color of the mix turns to dark-green the reaction becomes vigorous; the temperature at this stage depends on the Ln component, and lies above 300 °C. The optimum reaction temperature refers to the moment when the color of the liquid phase changes into dark greenish-blue. It is important to estimate this temperature (in a preliminary heating test) in order to improve the product yield. After the reaction is finished and the reactor cooled down to room temperature, the dark navy-blue crude product is removed from the ampoule, dissolved in dichloromethane and filtered. It is recommended to subject the filtrate to a preliminary chromatography process using a column filled with basic Al_2_O_3_ and washed with dichloromethane. After evaporating the solvent from the collected eluate the dry product is extracted in a conical laboratory flask by small volumes of DMF until the extract still contains certain amounts of the synthesized LnPc_2_-*R*_8_ complex, which could be determined from the UV-Vis spectra measured for the individual extracts. Further purification follows by a column chromatography performed on a silica gel filling, washed with DMF. A multiple process monitored by UV-Vis measurements is required to attain a high-purity product. The pure product yields obtained so far in our laboratory depend on the synthesized complex and usually fall in the range of 30–50% of the theoretical value. Quality of the final product could be evaluated based on the results provided by MALDI-ToF, UV-Vis and FTIR analysis.

### 3.3. Photostability Determination

Solutions of the tested complexes were prepared to allow initial absorbance values of about 1.5, corresponding to molar concentrations of the order of 10^−5^, independent on the complex. In this work, neither the solvent nor the solutions were de-aerated prior to photolysis. 4 mL of such solution was irradiated in a typical 1 cm quartz cuvette placed in a thermostated cuvette holder, at constant T = 20 °C, using UV radiation with λ_max_ at 352 nm and intensity of 300 μW/cm^2^ measured at the cell wall facing illumination. The reaction progress was monitored through a period of 360 min by time-correlated electronic absorption spectra acquired in the λ range 190–1100 nm. The spectra were analyzed using the Jasco Spectra Manager V.2 software. Kinetic curves referring to the photolysis reaction were determined for the B and Q-band absorption maxima of the relevant spectra and the effective rate constants (*k_e_*) were computed using specialized analytical software (Curve Expert v. 1.3).

## 4. Conclusions

The novel lanthanide bis-phthalocyanines peripherally functionalized by pentadecylphenoxy-groups represent a class of technologically interesting materials. They display some very important features of possible practical significance, such as probable discotic liquid crystalline nature expressed by a quasi-liquid consistency at room temperature, affinity for lipophilic media, considerable solubility in organic solvents and reasonable UV-photostability. Since these complexes are completely new materials further detailed investigations are necessary to assess their practical usefulness for high-tech applications.

## Supplementary Material

Supplementary materials can be accessed at: http://www.mdpi.com/1420-3049/17/9/10738/s1.
