# Supplementary material for: Novel Lipophilic Lanthanide Bis-Phthalocyanines Functionalized by Pentadecylphenoxy Groups: Synthesis, Characterization and UV-Photostability
Source: Molecules. 2012 Sep 7;17(9):10738–53. doi: 10.3390/molecules170910738 (PMC6268473; doi:10.3390/molecules170910738)
Supplement: Supplementary file 1 [file molecules-17-10738-s001.pdf]

Supplementary Information

Novel Lipophilic Lanthanide Bis-Phthalocyanines Functionalized by Pentadecylphenoxy Groups: Synthesis, Characterization and UV-Photostability

Rudolf Słota [1,*], Gabriela Dyrda [1], Maria Hofer [1], Giuseppe Mele [2], Ermelinda Bloise [2] and Roberta del Sole [2]

[1] Faculty of Chemistry, Opole University, ul. Oleska 48, 45-052 Opole, Poland
[2] Department of Engineering for Innovation, University of Salento, Via Arnesano, 73100 Lecce, Italy

* Author to whom correspondence should be addressed; E-Mail: rslota@uni.opole.pl;
Tel.: +48-77-452-7131; Fax: +48-77-452-7101.

1. Infrared Spectra

Basically, the spectra of all studied $LnPc_2$-R_8 compounds, measured in the infrared range 400–4000 cm^{-1}, display a similar general pattern as that of the europium complex shown below.

Figure S1. Effect of pentadecylphenoxy-substitution in $EuPc_2$ displayed in the FTIR spectra (note the x-axis has been broken between 1700 and 2700 cm^{-1} for brevity).

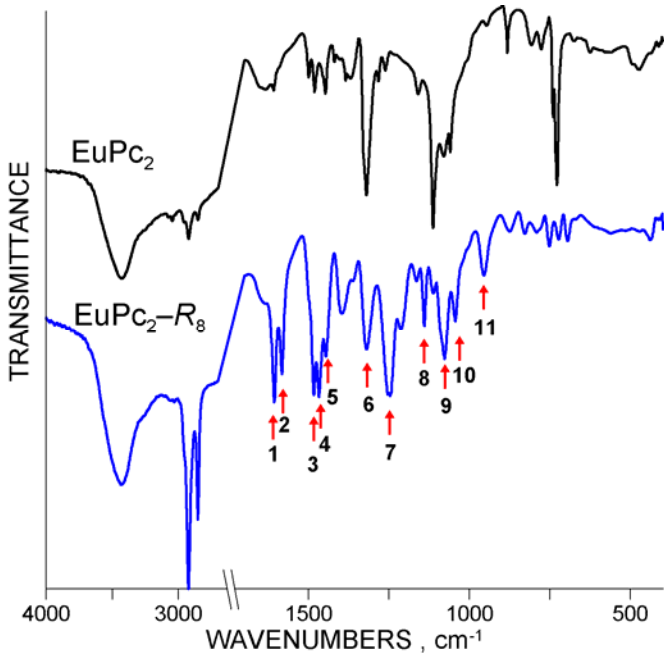

Table S1. Vibration frequencies (cm^{-1}) registered in the FTIR spectra of $LnPc_2$-R_8 corresponding to the numbers assigned to the chosen bands in Figure S1.

	**Wavenumbers, cm^{-1}**										
	**1**	**2**	**3**	**4**	**5**	**6**	**7**	**8**	**9**	**10**	**11**
Pr	1606	1583	1483	1467	1445	1322	1247	1142	1078	1044	962
Nd	1606	1583	1480	1467	1446	1323	1253	1141	1079	1043	957
Eu	1606	1582	1483	1467	1446	1320	1247	1140	1077	1044	955
Gd	1605	1582	1484	1466	1445	1320	1246	1141	1077	1044	957
Tb	1605	1582	1483	1467	1445	1322	1246	1142	1078	1044	960
Dy	1605	1582	1483	1466	1444	1324	1246	1141	1079	1044	960
Ho	1605	1583	1484	1467	1446	1324	1247	1142	1078	1045	960
Er	1605	1583	1483	1467	1445	1325	1246	1142	1078	1045	962
Tm	1605	1583	1483	1467	1445	1325	1247	1143	1080	1047	963
Yb	1605	1583	1483	1467	1445	1326	1247	1143	1080	1048	963
Lu	1606	1583	1484	1467	1446	1324	1247	1141	1080	1049	959

The IR spectra represent a very complex layout of absorption bands due to diverse coupling effects produced by the oscillating bonding system of the both phthalocyanine macrocycles. Distinct effect of the peripheral substitution is particularly pronounced in the following ranges: 2850–3000, 1582 (peak No. 2), 1445–1483 (No. 3–5), and 1247–1324 cm^{-1} (No. 6,7), Figure S1. Incidentally, these bands directly confirm the presence of pentadecylphenoxy-groups attached to the phthalocyanine moiety.

Unlike in the UV-Vis spectra, no general trend related to the individual lanthanide metals has been found (see Table S1). Characteristic metal-sensitive stretching vibrations of the coupled π-electronic C-N bonding system for most of mono-metallophthalocyanines are observed at about 1050–1090 cm^{-1}. For non-substituted $LnPc_2$'s this range lies between 1060 and 1080 cm^{-1} showing insignificant susceptibility to the Ln metal. This frequency range corresponds to 1043–1080 cm^{-1} (No. 9,10) in $LnPc_2$-R_8. Interestingly, the small shift (17 cm^{-1}) of the lower frequency value resembles somewhat the shift in UV-Vis spectra reported in Figure 6 (main text).

2. Fluorescence Emission Spectra

The novel $LnPc_2$-R_8 complexes and H_2Pc-R_4 excited by UV radiation show visible photonic emission both in the blue and red range of the visible spectrum. Under constant illumination performed during the photostability tests (λ = 352 nm) the $LnPc_2$-R_8's evidently displayed some pale-blue emission whereas for H_2Pc-R_4 it was a typical and distinctly red fluorescence, both of which could be observed. Diverse types of emission spectra registered for the studied compounds in DMF (and in CH_2Cl_2 for the H_2Pc derivative) have been shown in Figure S2 and the λ values related to the appropriate bands are reported in Table S2. Complexes of Er and Tm were not investigated.

Figure S2. Fluorescence emission spectra-types representative for $LnPc_2$-R_8 (in DMF) and H_2Pc-R_4 (in dichloromethane). Excitation (λ_{exc}) and emission (λ_1, λ_2) wavelength values have been shown in Table S2.

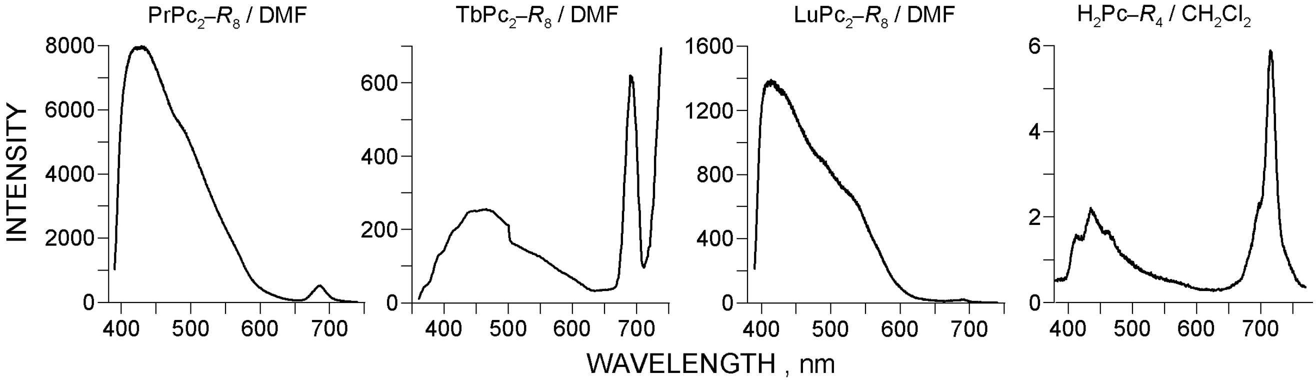

Table S2. Emission wavelength values determined for $LnPc_2$-R_8 (in DMF) and H_2Pc-R_4 (in dichloromethane); *a*—no red emission observed; [s] strong, [w] weak, [vw] very weak.

	Pr	Nd	Eu	Gd	Tb	Dy	Ho	Yb	Lu	H_2Pc
λ_{exc}	342	342	327	334	341	344	343	341	342	342
λ_1	423 [s]	456 [s]	457 [w]	450 [s]	456 [w]	554 [w]	435 [s]	407 [w]	410 [s]	440 [w]
λ_2	694 [w]	685 [w]	690 [s]	684 [w]	690 [s]	692 [s]	*a*	693 [s]	690 [vw]	714 [s]

Although the particular systems reveal no general relation to the Ln atom, certain interesting observation emerges from the analyzed spectra. Namely, some of the compounds showing strong blue emission (for Pr, Nd and Gd) have been found the least resistant to UV illumination (in DMF) whereas those with intensive red fluorescence (e.g., for Tb, Dy and Yb) displayed significantly higher photostability (discussed in section 2.6). This however is not a rule and the registered emission spectra reflect the individual physicochemical nature of the $LnPc_2$-R_8 complexes explored.

3. Elemental Analysis

The theoretically calculated chemical compositions (% mass) for the range of $LnPc_2$-R_8 are as follows :

$PrPc_2$-R_8 : C 77.6 H 8.7 N 6.2 O 3.6 Ln 3.9
$LuPc_2$-R_8 : C 76.8 H 8.6 N 6.2 O 3.5 Ln 4.8

The spread of analytical values determined for C, H and N in the studied compounds was:

C 73.4–77.0 H 7.9–8.4 N 5.3–5.7

The best-consistent result was obtained for $NdPc_2$-R_8 (and H_2Pc-R_4) :

$NdPc_2$-R_8 : C 75.0 H 8.2 N 5.5
H_2Pc-R_4 : calc. C 80.6 H 9.2 N 6.5 O 3.5
anal. C 78.5 H 8.8 N 6.2 ---

The analytical data acquisited for the N content (depending on the $LnPc_2$-R_8 compound) appeared to be more or less out of the expected (and to-be-accepted) range, due to unknown reason, so far. This issue will be explored in detail in a separate project.

Besides, it is worth to mention, that similar "problems" concerning the analytical results for the N content in metallophthalocyanines with octa-alkyl-substituted macrocycles may be found elsewhere, e.g., 9.6% less of the expected value shown for a Lu sandwich complex [13] or 11.4% more in the case of a Cu mono-complex (Piechocki et al., *J.Am.Chem.Soc.* **1982**, *104*, p.5246).

Nevertheless, the molecular identity of the novel $LnPc_2$-R_8 complexes reported in this work has been sufficiently well confirmed by the MALDI-ToF and UV-Vis spectra.

4. MALDI-ToF Results

Table S4. Mass spectra m/z results (MALDI-ToF) acquired for the novel bis-phthalocyanines $LnPc_2$-R_8 (no data available for Tb, Er, Tm) and the metal-free base phthalocyanine, and the calculated molecular mass (M).

Compound	M (calc.)	*m/z* (exp.)
$H_2Pc – R_4$	1726	1722–1726
$PrPc_2 – R_8$	3589	3583–3590
$NdPc_2 – R_8$	3593	3585–3593
$EuPc_2 – R_8$	3600	3592–3600
$GdPc_2 – R_8$	3605	3599–3604
$DyPc_2 – R_8$	3610	3604–3610
$HoPc_2 – R_8$	3613	3607–3612
$YbPc_2 – R_8$	3621	3617–3622
$LuPc_2 – R_8$	3623	3618–3623
